# Microstructure Characterization and Wear-Resistant Properties Evaluation of an Intermetallic Composite in Ni–Mo–Si System

**DOI:** 10.3390/ma10020130

**Published:** 2017-02-04

**Authors:** Boyuan Huang, Chunyan Song, Yang Liu, Yongliang Gui

**Affiliations:** 1 School of Materials Science and Engineering, Southwest Petroleum University, Chengdu 610500, China; simonsjz@163.com; 2 School of Metallurgy and Energy, North China University of Science and Technology, Tangshan 063009, China; 3 School of Mechanical Engineering, University of Science and Technology Beijing, Beijing 100083, China; liuyang_ustb_1988@163.com

**Keywords:** intermetallic compound, microstructure, wear-resistance, abrasive wear

## Abstract

Intermetallic compounds have been studied for their potential application as structural wear materials or coatings on engineering steels. In the present work, a newly designed intermetallic composite in a Ni–Mo–Si system was fabricated by arc-melting process with commercially pure metal powders as starting materials. The chemical composition of this intermetallic composite is 45Ni–40Mo–15Si (at %), selected according to the ternary alloy diagram. The microstructure was characterized using optical microscopy (OM), scanning electron microscopy (SEM), X-ray diffraction (XRD), and energy dispersive spectroscopy (EDS), and the wear-resistant properties at room temperature were evaluated under different wear test conditions. Microstructure characterization showed that the composite has a dense and uniform microstructure. XRD results showed that the intermetallic composite is constituted by a binary intermetallic compound NiMo and a ternary Mo_2_Ni_3_Si metal silicide phase. Wear test results indicated that the intermetallic composite has an excellent wear-resistance at room-temperature, which is attributed to the high hardness and strong atomic bonding of constituent phases NiMo and Mo_2_Ni_3_Si.

## 1. Introduction

It is more and more challenging for traditional engineering steel materials to satisfy the gradually growing performance requirements for wear-resistant materials which service in serious environments [1,2,3]. The research and development of new wear materials is driven strongly by the demands of higher and higher operating stresses and temperatures in mechanical moving components. From the tribological point of view, the combined metallic and covalent bond guarantees intermetallic compounds outstanding adhesive wear-resistance, and the high hardness and anomalous hardness–temperature relation provides excellent abrasive wear-resistant properties [4,5]. Increasing attention is given to intermetallic compounds because of their potential to be used as wear-resistant materials or coating candidates [6,7,8,9].

As a binary intermetallic compound, NiMo was given more interest for its thermodynamic properties in the past couple of years [10,11,12]. Little attention was focused on its mechanical properties, especially on wear-resistance [13,14]. It is a fact that monolithic intermetallic compounds are extremely brittle for structural application. Compared to monolithic intermetallic alloys, multi-component and multi-phase intermetallic composites could have a better combination of toughness and strength. Great effort has been made to further improve the comprehensive properties of intermetallic compounds and then promote their industrial applications [15,16,17,18].

In Ni–Mo–Si ternary system—metal silicide Mo_2_Ni_3_Si—a Laves phase with hP12 MgZn_2_-type crystal lattice has been widely reported to be a good wear-resistant phase in many intermetallic composites [19,20,21], e.g., Mo-toughened Mo_2_Ni_3_Si alloy, Mo_2_Ni_3_Si/NiSi, γ-Ni toughened Mo_2_Ni_3_Si etc. Furthermore, ternary metal silicides are regarded to have better toughness in comparison to the binary metal silicides as well as keeping the inherent high hardness and strong interatomic bonds [22]. Accordingly, the formation of metal silicide in a Ni–Mo–Si ternary system resulting from the addition of Si may be beneficial for the properties of intermetallic compounds by offering good creep resistance, low density, and wear-resistance [23,24]. Based on the previous research results, compared to the single Mo or γ-Ni phase-toughened Mo_2_Ni_3_Si, binary intermetallic compound NiMo-toughened Mo_2_Ni_3_Si composite displays better combination of strength and toughness for wear applications. The formation of some eutectic structures is also beneficial to the improvement of the toughness of intermetallic composites.

Among all reinforcing technologies, “in-situ” incorporation has proved to be an effective and practical approach [14,25,26,27]. In the present work, a new intermetallic composite in a Ni–Mo–Si ternary system was designed based on the concept of the “in-situ” formation of multi-phase intermetallic composite and fabricated successfully using arc-melting method, which has a microstructure of binary intermetallic NiMo and ternary metal silicide Mo_2_Ni_3_Si. The purpose is to clarify the microstructure and wear-resistance of NiMo/Mo_2_Ni_3_Sicomposites with different Si content in chemical composition and some eutectic structure in microstructure. The dry-sliding wear and abrasive wear-resistant properties of the new intermetallic composite were evaluated at ambient temperatures. The worn surface morphologies and wear debris collected after wear tests were examined by scanning electron microscope (SEM).

## 2. Experimental Procedures

The Ni–Mo–Si ternary system intermetallic composite ingots were manufactured by the arc-melting process with the commercially pure powders of Mo (99.9%), Ni (99.5%), and Si (99.96%) as the starting materials. The nominal chemical composition of the intermetallic composite is 45Ni–40Mo–15Si (at %), which was selected in the isothermal section of the Ni–Mo–Si ternary phase diagram at 1000 °C. To achieve the Ni–Mo–Si intermetallic composite with homogeneous microstructure and property, each ingot was re-melted three times in a different direction. More than 20 ingots were prepared for the microstructure characterization and wear tests. The detailed fabricating parameters for the arc melting process—taken according to previous works—are optimized for an electric current of 300 A, voltage of 11–12 V, and the argon pressure of 62–67 kPa.

The microstructure of the Ni–Mo–Si ternary system intermetallic composite was characterized by an invert-type optical microscope (OM, Carl Zeiss Light Microscope GmbH, Göttingen, Germany) and scanning electron microscope (SEM, KYKY Technology Development Ltd., Beijing, China). The phases were identified by the X-ray diffraction (XRD, Rigaku Corporation, Tokyo, Japan) method using a Rigaku D/max 2200 pc automatic X-ray diffractometer with Cu target Kα radiation and a scanning rate of 5°/min. Energy dispersive spectroscopy (EDS, KYKY Technology Development Ltd., Beijing, China) equipped on SEM was used to analyze the chemical composition of each phase in the Ni–Mo–Si ternary system intermetallic composite. The average hardness of the intermetallic composite was measured in a micro-hardness indenter with the load-dwell time of 15 s and the compression load of 500 g.

**Figure 1:**
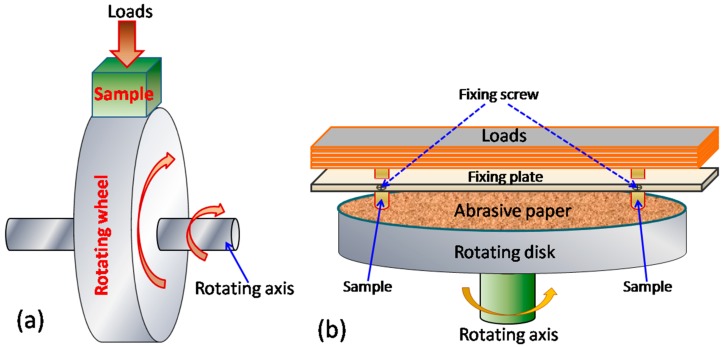
Schematic illustration of (**a**) block-on-wheel dry sliding wear and (**b**) abrasive wear tester.

The dry sliding wear-resistance of the 45Ni–40Mo–15Si intermetallic composite was evaluated on a block-on-wheel dry sliding wear tester at room temperature. The block-like specimen (10 mm × 10 mm × 10 mm in size) cut off from the ingots was pressed under an applied test load of 147 N against the outer periphery surface of a hardened 0.45% C steel (HRC53) rotating wheel. The relative sliding speed of 0.9 m/s and the total wear sliding distance is 3186 m. Abrasive wear behavior was examined by using an abrasive wear tester (shown in Figure 1) by applying 49 N load at ambient temperature. Commercial 320 grit SiC-silicon carbide grinding paper stuck on the rotating disk was selected as an abrasive particle with the size of approximately 40 µm. The test samples were 6 mm in diameter. The abrasive wear test parameters are as follows: sliding speed 0.3 m/s, wear test time 1 min and total wear sliding distance 18 m. The hardened 1.0% C–1.5% Cr bearing steel (HRC63) and high-chromium cast-iron (HRC65) were selected as the reference material for the dry sliding wear and abrasive wear tests, respectively. Wear mass loss was used to rank the wear-resistant property of the test materials (the lower the wear mass loss, the higher the wear-resistance). The worn surface and debris morphologies were examined using SEM, and the chemical compositions of debris were analyzed by EDS.

## 3. Results and Discussion

**Figure 2:**
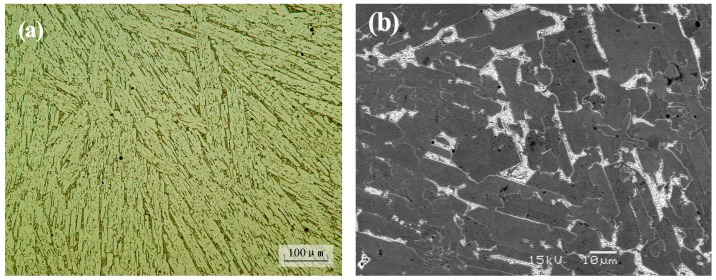
(**a**) Optical microscope (OM) and (**b**) scanning electron microscope (SEM) microscopy showing the typical microstructure morphologies of the Ni–Mo–Si ternary intermetallic composite.

**Figure 3:**
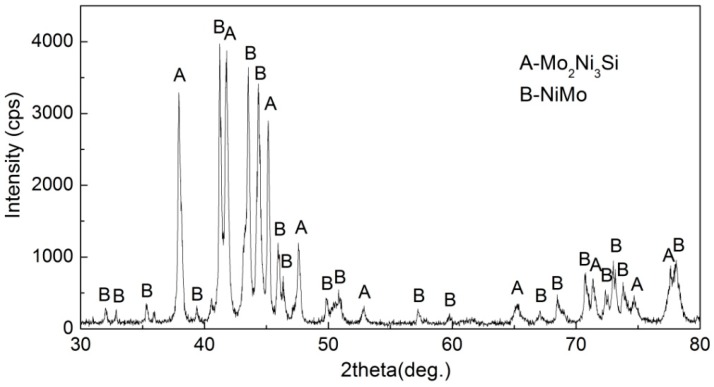
X-ray diffraction (XRD) profiles of the Ni–Mo–Si intermetallic composite.

The arc-melted Ni–Mo–Si ternary system intermetallic composite has a dense and uniform microstructure which consists of primary dendrites, interdendritic matrix, and remained eutectic structure, as shown in Figure 2. Results of XRD (shown in Figure 3) and EDS analysis indicate that the primary dendrites are the NiMo binary intermetallic compound, the interdendritic matrix is ternary metal silicide Mo_2_Ni_3_Si, and the eutectic structure is the NiMo/Mo_2_Ni_3_Si. The 45Ni–40Mo–15Si intermetallic composite has an average hardness of approximately 920 HV (HRC67.5) and uniform hardness distribution within the composite.

**Figure 4:**
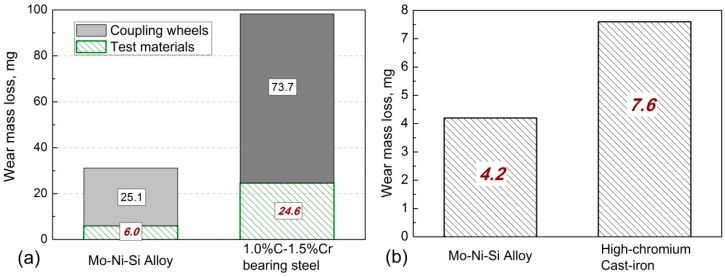
Wear mass loss of the Ni–Mo–Si ternary intermetallic composite and reference test materials under (**a**) room temperature dry-sliding and (**b**) abrasive wear conditions.

Figure 4 shows wear mass loss of the 45Ni–40Mo–15Si ternary intermetallic composite and reference materials under different wear conditions. Wear mass loss of the intermetallic composite is significantly less than that of the reference material, not only under dry-sliding wear, but also under abrasive wear conditions at ambient temperature. The relative wear-resistance (the ratio of wear mass loss of the 45Ni–40Mo–15Si intermetallic composite to that of the comparison material) of the intermetallic composite was up to four times higher than the hardened 1.0% C–1.5% Cr bearing steel at block-on-wheel wear environments with the applied load of 147 N. Several wear test results of different materials are listed in Table 1. These results demonstrate that the 45Ni–40Mo–15Si ternary intermetallic composite constituted by NiMo and Mo_2_Ni_3_Si intermetallic phases, exhibits better wear-resistant properties than traditional engineering steel materials.

Moreover, as shown in Figure 4a, wear mass loss of the coupling hardened 0.45% C steel wheel with the intermetallic composite as a wear test material is much lower that with the reference 1.0% C–1.5% Cr bearing steel as wear test material. Tribologically, the behavior of metallic materials in wear conditions is highly dependent on the chemical affinity of decoupled wear elements. The reference steel material has the same metallic bond with the wear coupling wheel, which led to a strong chemical affinity and serious wear loss of both wear test materials and the coupling wheel. The covalent-dominant atomic bonding attributes of NiMo and Mo_2_Ni_3_Si phases in the 45Ni–40Mo–15Si ternary intermetallic composite led to poor chemical affinity with its wear steel counterpart.

**Figure 5:**
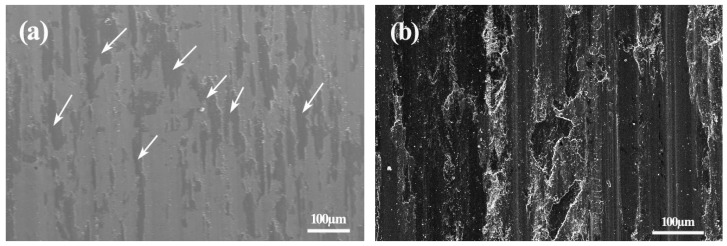
SEM micrographs showing the worn surface morphologies of (**a**) the Ni–Mo–Si ternary intermetallic composite and (**b**) hardened 0.45% C steel wheel counterpart under room temperature dry-sliding wear conditions.

The worn surface of the 45Ni–40Mo–15Si ternary intermetallic composite after the dry-sliding wear test was very smooth without any visible wear grooves, scratches, or adhesion patches (as shown in Figure 5a), due to its high hardness and strong atomic bonds. As indicated in Figure 5b, the worn surface of the counterpart hardened 0.45% C steel wheel was also smooth, with only shallow grooves but no adhesion features visible because the poor chemical affinity between wear coupling materials (block-like intermetallic composite and steel wheel) effectively prohibited the sliding surface from metallic adhesion.

As the arrows in Figure 5 indicate, some cover layers were formed on the worn surface during the wear test process. EDS examination results revealed that the chemical composition of the cover layers was approximately Fe55%Ni12%Mo9%Si4%O20%, which is highly consistent with the elemental constituents of wear debris collected after wear tests. It could be concluded that the cover layers are most likely the rolling-consolidated products of tiny wear powders. Undoubtedly, the cover layers contribute to improve the wear-resistance of both the tested intermetallic composite and coupling steel wheel. On the one hand, the cover layers on the contact surface could prevent the intermetallic composite from direct touch with the mating wear wheel, which reduces the adhesion wear. On the other hand, the wear attacks from the contact surface of the rotating steel wheel were effectively avoided, as some partial contact surfaces of the test intermetallic composite were not exposed to the wear mating wheel.

**Figure 6:**
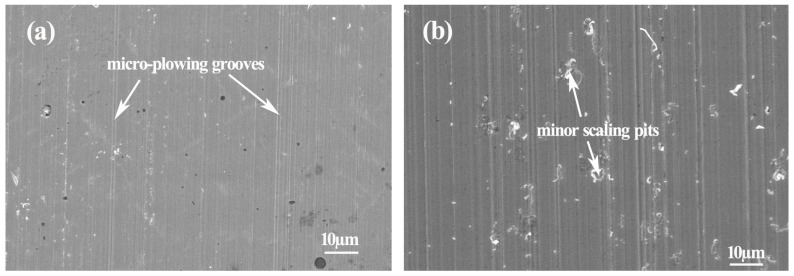
Worn surface morphologies of (**a**) the Ni–Mo–Si ternary intermetallic composite and (**b**) high-chromium cast-iron after abrasive wear for 18 m at room temperature.

The worn surface morphologies of the intermetallic composite and high-chromium cast-iron after abrasive wear are illustrated in Figure 6. As the arrows indicate, micro-cutting and plowing grooves are observed on the worn surface of the intermetallic composite, while minor scaling areas and pits can be discovered beside plowing grooves on the worn surface of the high-chromium cast-iron (see Figure 6b).

Generally speaking, tribological behaviors depend on the metallographic structure of the service wear materials. The Ni–Mo–Si ternary intermetallic composite seems to be ideally suited for wear application, and is promising in dry-sliding and abrasive wear fields as wear materials or coatings. In resisting abrasive wear attacks, the constitutional phases—both binary intermetallic compound NiMo and ternary metal silicide Mo_2_Ni_3_Si—play a dominant role in resisting abrasive adhesive wear resulting from the inherent high hardness. In the dry-sliding wear process, the strong covalent-dominant atomic bondings of NiMo and Mo_2_Ni_3_Si phases endow the 45Mo–40Ni–15Si composite excellent resistant capability to adhesive wear damages, and prevented test samples from plastic deformation, adhesion, and materials-transferring. This is evidenced by the smooth worn surface of both block-like sample and steel wheel counterpart.

**Figure 7:**
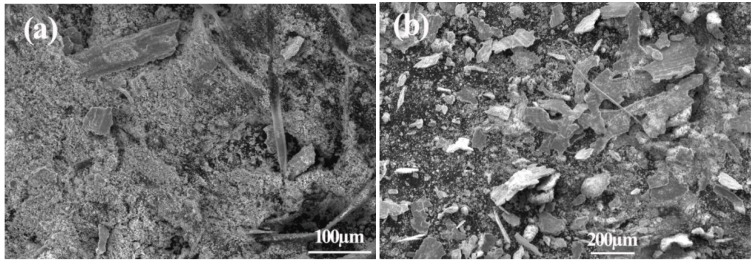
SEM micrographs showing the wear debris of (**a**) the Ni–Mo–Si ternary intermetallic composite and (**b**) hardened 1.0% C–1.5% Cr bearing steel under room temperature dry-sliding wear conditions.

The wear debris of the 45Ni–40Mo–15Si ternary intermetallic composite collected after each dry-sliding wear test displayed a size distribution from tiny powder, filament-like debris, to flake-like fragments, as shown in Figure 7a. Careful examination revealed that the filament-like debris was up to 300 µm in length, and the flake-like fragments were up to 200 µm × 70 µm in size. EDS examination revealed that the chemical composition of tiny wear powders was Fe57%Ni11%Mo7%Si4%O21%, which indicates that they are mixed metal oxides and mainly from the coupling steel wheel because of the high Fe content. The filament-like debris are cutting products from rotating steel wheel, and the flake-like fragments are an agglomeration of tiny debris powders. In comparison, wear debris of the reference hardened 1.0% C–1.5% Cr bearing steel after dry-sliding wear tests was large, as shown in Figure 7b. The wire-like wear debris was the adhesion product originating from either the block-like test sample or coupling wheel.

In summary, the relatively smooth worn surface without obvious plastic deformation and selective wear confirms that the 45Ni–40Mo–15Si intermetallic composite kept high strength and high hardness in the wear process. The above results display that the Ni–Mo–Si intermetallic composite has outstanding wear-resistance under dry sliding wear and abrasive wear environments, which indicates that the composite would be a potential candidate material in wear applications.

## 4. Conclusions

(1) A wear-resistant 45Ni–40Mo–15Si intermetallic composite was designed based on the Ni–Mo–Si ternary alloy diagram and was manufactured successfully by an arc-melting route with pure nickel, molybdenum, and silicon powder blends as starting materials.
(2) Microstructural characterization revealed that the new intermetallic composite had a dense and uniform microstructure, and the main constituent phases were binary intermetallic compound NiMo and ternary metal silicide Mo_2_Ni_3_Si.
(3) The Ni–Mo–Si ternary intermetallic composite exhibited better wear-resistant properties compared to traditional engineering steel materials under the same room-temperature dry-sliding and abrasive wear test conditions, which implies its potential in various wear environments.

## Figures and Tables

**Table 1:** Wear test results of different materials under block-on-wheel dry-sliding wear conditions with 147 N applied load at room temperature.

Test Materials	Wear Mass Loss (mg)		Wear Volume Loss (mm^3^)	
	Sample	Counterpart	Sample	Counterpart
45Ni–40Mo–15Si	6.0	24.6	0.66	3.14
Hardened 1.0% C–1.5% Cr Bearing Steel	25.1	73.7	3.21	9.40
Hardened 0.45% C Steel	39.4	135.7	5.03	17.32
Austenitic 1Cr18Ni9Ti Stainless Steel	92.2	266.3	11.74	33.98
